# Therapy of Fabry disease with pharmacological chaperones: from in silico predictions to in vitro tests

**DOI:** 10.1186/1750-1172-6-66

**Published:** 2011-10-17

**Authors:** Giuseppina Andreotti, Valentina Citro, Agostina De Crescenzo, Pierangelo Orlando, Marco Cammisa, Antonella Correra, Maria Vittoria Cubellis

**Affiliations:** 1 Istituto di Chimica Biomolecolare - CNR, Pozzuoli, Italy; 2 Institute of Genetics and Biophysics 'A. Buzzati Traverso,' CNR, Naples, Italy; 3 Dipartimento di Scienze Ambientali, Seconda Università di Napoli, Caserta, Italy; 4 Institute of Protein Biochemistry-CNR, Napoli, Italy; 5 Dipartimento di Biologia Strutturale e Funzionale, Università Federico II, Napoli, Italy; 6 Istituto di Biostrutture e Bioimmagini-CNR, Napoli, Italy

## Abstract

**Background:**

Fabry disease is a rare disorder caused by a large variety of mutations in the gene encoding lysosomal alpha-galactosidase. Many of these mutations are unique to individual families. Fabry disease can be treated with enzyme replacement therapy, but a promising novel strategy relies on small molecules, so called "pharmacological chaperones", which can be administered orally. Unfortunately only 42% of genotypes respond to pharmacological chaperones.

**Results:**

A procedure to predict which genotypes responsive to pharmacological chaperones in Fabry disease has been recently proposed. The method uses a position-specific substitution matrix to score the mutations. Using this method, we have screened public databases for predictable responsive cases and selected nine representative mutations as yet untested with pharmacological chaperones. Mutant lysosomal alpha galactosidases were produced by site directed mutagenesis and expressed in mammalian cells. Seven out of nine mutations responded to pharmacological chaperones. Nineteen other mutations that were tested with pharmacological chaperones, but were not included in the training of the predictive method, were gathered from literature and analyzed in silico. In this set all five mutations predicted to be positive were responsive to pharmacological chaperones, bringing the percentage of responsive mutations among those predicted to be positive and not used to train the classifier to 86% (12/14). This figure differs significantly from the percentage of responsive cases observed among all the Fabry mutants tested so far.

**Conclusions:**

In this paper we provide experimental support to an "in silico" method designed to predict missense mutations in the gene encoding lysosomal alpha galactosidase responsive to pharmacological chaperones. We demonstrated that responsive mutations can be predicted with a low percentage of false positive cases. Most of the mutations tested to validate the method were described in the literature as associated to classic or mild classic phenotype. The analysis can provide a guideline for the therapy with pharmacological chaperones supported by experimental results obtained in vitro. We are aware that our results were obtained in vitro and cannot be translated straightforwardly into benefit for patients, but need to be validated by clinical trials.

## Background

Fabry Disease (FD) [ORPHANET: orpha324, OMIM: 30150] is a pan-ethnic disorder caused by mutations in the gene encoding lysosomal alpha galactosidase [HGNC:GLA; UNIPROT: AGAL_HUMAN,] (for a review [1]). The classic form of the disease is characterized by angiokeratomas, acroparesthesia, hypohidrosis, corneal opacity in childhood or adolescence, and progressive vascular disease of the heart, kidneys and central nervous system[2]. Although FD follows X-linked inheritance, heterozygous females can be symptomatic [3]. The reported incidence of FD in the general population ranges from 1 in 476,000 [4] to 1 in 117,000 [5]; however this may well underestimate the true proportion of affected people. Indeed, in a large screening of Italian male newborns revealed an occurrence of mutations in the gene encoding AGAL as high as 1 in 3100 [6]. FD may be under-diagnosed because its phenotypic manifestations are heterogeneous and partially coincident with those of common diseases, and because many patients develop symptoms in adolescence or even later. An indication of the multiplicity of mutations is given by the fact that 344 missense/non sense mutations of the AGAL gene have been recorded in the public version of HGMD [7] and even more in the professional version of the same database. Most of these mutations are "private", that is observed in only one family.

This heterogeneity of genotypes explains only part of the heterogeneity observed in phenotypes because clinical phenotype, age of onset and course of Fabry disease are highly variable, even within the same family [8]. At present the treatments of Fabry disease are symptomatic and life-long. Enzyme replacement (ERT) has already been introduced into medical practice and is considered " the clinical gold standard". This therapy requires intravenous infusions of purified AGAL produced by genetically engineered cell lines every 2 weeks [9]. Treatment with pharmacological chaperones (PCs), in particular 1-deoxy-galactonojirimycin is in phase three clinical trials. 1-deoxy-galactonojirimycin, also known as DGJ, migalastat hydrochloride or AT1001 is an imino sugar that resembles galactose. The therapeutical approach with PCs relies on competitive inhibitors of AGAL that bind and stabilize the enzyme, increasing its total cellular levels, as shown in cultured cells and in vivo [10-12]. In theory PCs represent a useful alternative to ERT because they do not cause adverse immunological reactions and can be administered orally. In practice therapy with PCs is limited because only some mutations can be rescued by these drugs [13]. It has been observed that usually, but not necessarily, late-onset forms, as well as mutations that do not occur in the catalytic domain of AGAL, respond to 1-deoxy-galactonojirimycin.

We have recently proposed a method to predict which mutations in AGAL are responsive to 1-deoxy-galactonojirimycin. We have trained a classifier using published data concerning responsive and non responsive mutations [14-18] and evaluated 87% accuracy by cross validation. In this paper we extended this study by testing the method on mutations not included in the classifier training.

## Results

### Selection of mutations

Three-hundred AGAL mutant sequences from Uniprot/Swissprot [19] and HGMD [7] were analyzed with the method described previously [20]. This method relies on a Position Specific Substitution Matrix (PSSM) calculated from sequences homologous to AGAL. It assigns a score to a mutation taking into account the degree of conservation of the wild type amino acid in homologous sequences and the specific substitution that is introduced: if the new amino-acid is present in homologous species, a less negative score is obtained. The method was benchmarked and a threshold was set: scores equal or higher than -1 were associated with mutations responsive to 1-deoxy-galactonojirimycin with 87% accuracy (correctly predicted responsive mutations plus correctly predicted non responsive mutations divided by total number per 100).

Among the AGAL mutations present in HGMD [7] and as yet untested for their responsiveness to PC, we found 35 mutations scoring 0 or -1. We selected 4 representative mutations with score 0, and 5 representative mutations with score = -1 for further analysis.

This is a minimal set, but it is a good representation of the different types of mutations that can be encountered in AGAL. The set includes mutations occurring in different structural domains of the protein (TIM or beta), in different elements of secondary structure (alpha helix, beta strand, coil, polyproline II), at solvent exposed or non exposed sites. We also included mutations with different thermodynamic stability (assessed with MUPRO [21] or SDM [22,23]) and the rare case of a mutation affecting a non conserved residue of the active site (table 1). Mutant enzymes were produced by site directed mutagenesis and expressed transiently in COS-7 cells.

**Table 1:** Characteristics of the Fabry mutations tested by expression in COS-7 cells

Mutant: nucleotide	Amino acid	fold increase	PSSM	MUPRO	SDM	PSAmc	PSAsc	sec_stru	domain	Active site	Pheno type	ref
c.688G > A	p.A230T	2.1	0	-2.33	-0.48	4	64.8	P(5)	TIM	Yes	Classic	[24]
c.730G > C	p.D244H	3.3	-1	-1.74	0.379	18.6	48.7	A(11)	TIM	No	Classic	[31]
c.805G > A	p.V269M	2.5	0	-0.77	-0.212	3.2	0	O(12)	TIM	No	Classic	[28]
c.838C > A	p.Q280K	3.1	0	-1.00	-1.126	0	17.3	A(14)	TIM	No	Classic	[32]
c.898C > T	p.L300F	5.8	-1	-1.60	-0.732	9.9	0	O(6)	TIM	No	?	[30]
c.902G > C	p.R301P	3.7	-1	-1.68	-1.228	65.2	17.5	O(6)	TIM	No	Classic	[25]
c.928C > T	p.L310F	8.9	0	-1.34	-1.334	1.7	5.1	A(5)	TIM	No	Classic	[26]
c.1023A > C	p.E341D	2.2	-1	-0.88	-1.055	0	0	B(9)	beta	No	Classic	[27]
c.1228A > G	p.T410A	3.5	-1	-1.47	0.215	0	0	O(1)	beta	No	Variant	[29]

Mutations tested by transient transfection are listed together with their responsiveness to 1-deoxy-galactonojirimycin expressed as fold increase, the scores obtained by PSSM (assessment of conservation in homologous sequences), the scores obtained by SDM and MUPRO (assessment of the effect of mutations on thermodynamic stability), % main chain accessibility (PSAmc), % side chain accessibility (PSAsc), occurrence in A alpha-helix, B beta strand, P poly-proline II or O other (with the length of the secondary structure in which the mutation occur in brackets), occurrence in specific structural domains and in the active site. Severity in patients was deduced from literature and the reference paper is reported alongside.

### Activity of mutants with and without pharmacological treatment *in vitro*

**Figure 1:**
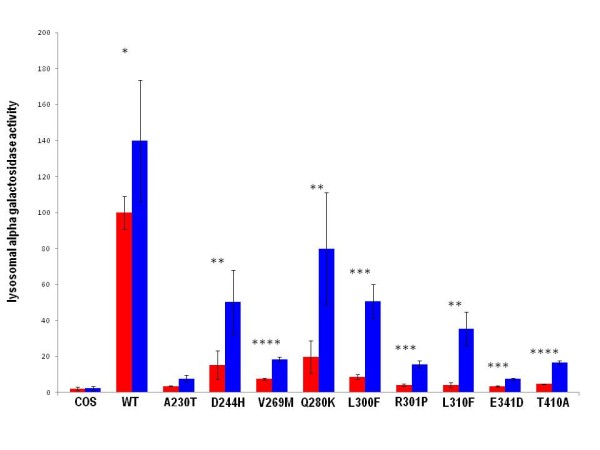
**Alpha-galactosidase activity resulting from 1-deoxy-galactonojirimycin administration in transfected COS-7 cells**. Four independent experiments performed in duplicate for 1-deoxy-galactonojirimycin (20 micromolar) administration in COS-7 transfections were performed. Values are expressed as percentage of COS-7 cells transfected with wild type. Standard deviations is indicated by bars and *differences *that are statistically significant are flagged by asterisks (p < 0.05:*; p < 0.01: **; p < 0.005:***; p < 0.001 :****)

Consistent with the clinical phenotype (classic FD) associated with mutations A230T [24], R301P [25], L310F [26], E341D [27] and V269M [28], the activity of the enzymes carrying these mutations was very close to the background i.e. the activity of COS-7 cells transfected with the void vector (figure 1). Four cases of hemizygotes affected with variable symptoms, ranging from severe to mild and with low to intermediate residual AGAL activity, were described for T410A [29]. This mutation, had a residual activity just above background when expressed in COS-7 cells. For L300F [30] we also measured residual activity above background, but no information is given on whether the associated phenotype of the patient was classic or mild. We measured residual activity above background for D244H [31] which was identified in a heterozygous female whose affected sons had the classic phenotype and for Q280K [32].

Most mutations associated to Fabry disease have been observed in single families and for this reason both the classification based on symptoms and the correlation with residual activity can be difficult.

The comparison of enzymatic activity of mutants with that of wild type expressed in COS-7 is summarized in figure 1; red bars indicate results obtained without the treatment with 1-deoxy-galactonojirimycin and blue bars indicate results obtained culturing the cells with 20 micromolar 1-deoxy-galactonojirimycin for 48 hours.

It is unsurprising that exposure to 1-deoxy-galactonojirimycin enhances alpha-galactosidase activity, even in the cells transfected with the wild type AGAL construct, since it was demonstrated, using scanning calorimetry, that wild type AGAL is stabilized by 1-deoxy-galactonojirimycin at both neutral and acidic pH [33]. Conditions that stabilize proteins, such as the presence of PCs, can increase the fraction of newly folded enzyme exported from the endoplasmic reticulum.

Seven out of nine mutations recovered 15% of wild type activity or more (figure 1), the exceptions being A230T and E341D. Although severity of Fabry disease correlates with the level of residual activity, it is difficult to determine the minimum level necessary to prevent the classic phenotype.

In order to set such a level we collected two groups of data from literature. The first group comprises 72 mutations whose residual activity has been assayed in fibroblasts derived from male patients [15], the second group comprises 21 mutations produced by site directed mutagenesis and assayed in COS cells [17,18]. The authors of the original papers provided the phenotype associated with each mutation. In both groups, mutations with a residual activity higher than 12% were associated with atypical/late phenotype, the only exception being E59K with 19 ± 3% residual activity [15]. This mutant has an abnormally high Km [2]; hence it might have a high residual activity at the saturating conditions which are generally used to assay enzymes in vitro, but not at the physiological concentrations of substrate present in the cell.

In consideration of the error associated with residual activity measures, we rounded by excess the minimum level sufficient to prevent classic phenotype to 15%. Our threshold was set quite cautiously, but does not differ significantly from that estimated by Fan and Ishii who stated "one would assume that residual enzyme activity greater than 10% of normal in hemizygote patients might be sufficient at reducing the majority of clinical symptoms" [34].

In women, the residual enzymatic activity differs from cell to cell due to X-inactivation and so values measured in serum might not correlate with the severity of the disease; nonetheless, raising residual activity above a minimal level in cells expressing the mutant allele, can still be beneficial for female patients.

The strongest response in terms of ratio between cellular activity obtained with or without treatment with 1-deoxy-galactonojirimycin was observed with L300F (table 1). Unfortunately in this case we were not able to deduce from the original paper the phenotype of the affected patients, but we learned from literature [15] that a mutation affecting the same site, L300P, is associated with classic phenotype and is responsive to 1-deoxy-galactonojirimycin with an even higher increase of activity.

### Western blot

**Figure 2:**
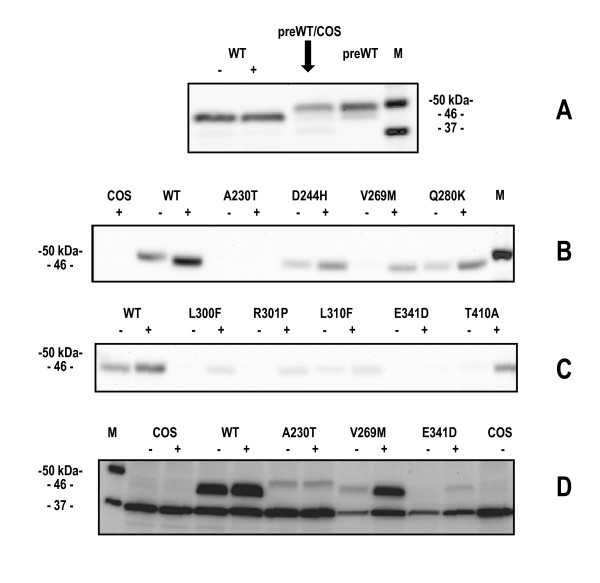
**Effect of 1-deoxy-galactonojirimycin on various mutant of alpha-galactosidase expressed in COS-7 cells**. Western blot analysis. Cells were cultured in the presence (+) or absence (-) of 20 uM 1-deoxy-galactonojirimycin for 2 days prior to western blot analysis. Panel A: WT-, COS-7 transfected with wild type AGAL (20 micrograms of cell extract); WT+, COS-7 transfected with wild type AGAL and treated with 20 micromolar 1-deoxy-galactonojirimycin (15 micrograms of cell extract); preWT/COS, COS-7 tranfected with void vector (20 micrograms) plus alpha-galactosidase precursor form (Fabrazyme^® ^20 nanograms), preWT, alpha-galactosidase precursor form (Fabrazyme^® ^20 nanograms); M, marker;. Panel B,C,D: COS-7 transfected with void vector (COS7), wild-type (WT) or mutant alpha-galactosidase vectors (10 micrograms) not treated (-) or treated with 1-deoxy-galactonojirimycin (+).

Human alpha-galactosidase is synthesized as a precursor of 50 kDa, but it is converted into a mature form of 46 kDa. A detailed analysis of Ishii and coworkers [2] demonstrated that when AGAL mutants are correctly processed into a 46 kDa form, they are also transferred into lysosomes. We first compared the migration of wild-type AGAL expressed in COS-7 cells, with and without treatment with PCs, to that of the precursor form (Fabrazyme^®^). In order to have bands of similar intensity, we loaded 20 micrograms of COS-7 extracts non treated with 1-deoxy-galactonojirimycin, 15 micrograms of COS-7 extract treated with 1-deoxy-galactonojirimycin (48 hours 20 micromolar), 20 micrograms of COS-7 extracts from cells transfected with void vector to which we added 20 nanograms of Fabrazyme^®^). and 20 nanograms of Fabrazyme^®^). AGAL bands were revealed by Western blot. As predicted, AGAL expressed in COS-7 migrated as 46 kDa band while the precursor migrated as 50 kDa band (figure 2 panel A). We then tested the migration of the mutants.

D244H, V269M, Q280K, L300F, R301P, L310F,T410 migrated as the wild type enzyme (figure 2 panel B and C) and upon treatment of cells with 1-deoxy-galactonojirimycin (48 hours 20 micromolar), the same protein band was observed with enhanced intensity. In figure 2 panel D the bands produced by those mutants displaying a low relative increase of activity upon treatment with the drug (fold increase <3 in table 1) were detected with longer acquisition time. A230T shows a faint band corresponding to the precursor form of AGAL which is not induced by the treatment with 1-deoxy-galactonojirimycin, V269M and E341D on the other hand, show bands corresponding to that of the mature form upon induction although the intensity of the band of the latter mutant is very weak.

The results of Western blot analysis demonstrates that treatment with the drug enhances the quantity of AGAL in the cell possibly stabilizing it and preventing its degradation.

### Analysis of mutants

We measured the ratio of the cellular activity of AGAL mutants treated or not treated with 1-deoxy-galactonojirimycin and expressed it as fold increase (table 1). We calculated structural and functional characteristics and PSSM score for each mutant and reported them together with the clinical phenotype obtained from literature (table 1). As previously explained, the mutations were chosen among those present in HDMG as representative cases with a relatively high score (0 or -1) obtained with the method based on PSSM construction.

:AGAL is a dimeric protein, each subunit being formed by a TIM barrel catalytic domain (res 32-325) and a small 8 stranded beta domain (res 326-429) whose function is not known [33]. We showed that occurrence in the TIM barrel catalytic domain does not prevent responsiveness to 1-deoxy-galactonojirimycin. This does not contradict the observation reported in literature [15] that mutations in the beta domain are mainly responsive, but reinforces the idea that mutations responsive to 1-deoxy-galactonojirimycin are widespread in the enzyme structure.

We listed structural features such as main chain and side chain accessibility of the affected site, type and length of the secondary structure where mutation occurs, the effect of the mutation on thermodynamic stability assessed with a method which rely on 3D-structure, such as SDM [22,23], or with a method which relies only on the sequence of the affected protein and the nature of the mutation, such as MUPRO [21].

All the mutants respond to 1-deoxy-galactonojirimycin in terms of fold increase (table 1), but if we consider the percentage of activity in the presence of 1-deoxy-galactonojirimycin compared to that of wild type, E341D and A230T might not recover enough activity to revert classic phenotype (figure 1). E341D is not conserved in homologous sequences and hence gets a high score by the PSSM, nonetheless it has a small response. An explanation exists for this since E341 occurs in a completely buried site and its mutation to aspartate is negatively scored by SDM [22,23], with a medium/severe adverse effect on thermodynamic stability (table 1). As demonstrated in our previous paper [20], SDM scores and solvent accessibility correlate with responsiveness; therefore co-occurrence of a low SDM score and low solvent accessibility explains the low response of E341D to the drug.

In contrast, A230 is exposed to solvent and its mutation to Threonine is mildly destabilizing.

Analysis of the enzyme active site explains why A230T responds very little to 1-deoxy-galactonojirimycin. A230 is not directly implicated in catalysis, but it is flanking D231, a proton donor that is essential for the hydrolysis of the substrate.

**Figure 3:**
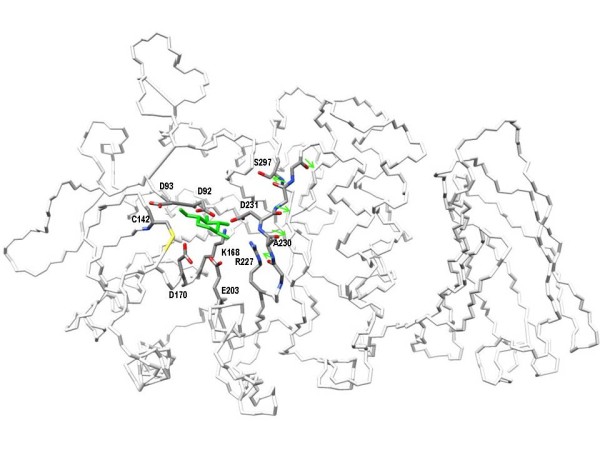
**The active site of human lysosomal alpha-galactosidase**. The active site of alpha-galactosidase is sketched as a wireframe coloured by atom type showing the side chains of residues which directly bind 1-deoxy-galactonojirimycin (D92, D93, D170, D231,E203, K168, R227), the side chains of C142, A230,S297 and the backbone of the peptide, ranging from 229 to 232, in poly-proline II helix conformation. Arrows run alongside carbonyl bonds. 1-deoxy-galactonojirimycin is coloured in green.

In addition, A230 belongs to a stretch of amino acids (229-232) that adopts a poly-proline II helix structure: this kind of secondary structure is quite rare in proteins and is often associated to special functions [35]. Figure 3 shows the active site of AGAL and the side chain of residues binding 1-deoxy-galactonojirimycin like D231, the backbone of aminoacids 229-232 and the side chain of A230 are shown. The characteristic three fold symmetry of the polyproline-helix can be seen from the spatial disposition of the carbonyl bonds. Although a direct proof could come only solving the X-ray structure of A230T, we can tentatively suggest that this secondary structure is affected by the mutation.

Close proximity to an essential residue and occurrence in a poly-proline helix explains why the mutation to Threonine results in a non responsive enzyme: mutations affecting the active site should be unable to bind the chaperone.

In this study we tested our method on mutations that had not been included into the training set and in particular on cases which our method predicted as responsive to the pharmacological therapy. Given that a therapy of general applicability (ERT) is already available, it is very important to prove that the percentage of false positive cases is low. We found that of nine AGAL mutants getting a PSSM score of 0 or -1 and expressed in cultured cells, seven responded to 1-deoxy-galactonojirimycin. In order to further validate the precision of our method, we looked in the literature for additional mutations which had been tested with 1-deoxy-galactonojirimycin, but not used in the training set of our method. We gathered 11 responsive and 8 non responsive cases and calculated their PSSM scores (table 2). In this set, the five mutations scoring -1 or 0 were all responsive, those scoring -2 were mostly (4/6) responsive, those scoring less than -2 were mostly (6/8) non responsive.

**Table 2:** Characteristics of the Fabry mutations gathered from literature

Mutant: nucleotide	aminoacid	PSSM	MUPRO	SDM	PSAmc	PSAsc	sec_stru	domain	ACT.SITE	resp	ref
c.58G > C	p.A20P	-2	-2.00	NA	NA	NA	NA	leader pep.	No	Yes	[2]
c.153G > A	p.M51I	-2	-0.51	0.414	0.0	27.1	O(15)	TIM	No	Yes	[41]
c.155G > A	p.C52Y	-5	-1.37	-1.582	29.5	27.3	O(15)	TIM	No	No	[14]
c.194G > C	p.S65T	-1	-0.85	0.330	0.0	25.5	O(15)	TIM	No	Yes	[42]
c.214A > G	p.M72V	-1	-0.27	-1.232	0.0	0.0	A(10)	TIM	No	Yes	[2]
c.426C > G	p.C142W	-7	-0.91	0.167	64.6	33.8	O(15)	TIM	Yes	No	[18]
c.436C > T	p.P146S	-3	-0.44	-0.983	1.4	19.5	O(15)	TIM	No	Yes	[2]
c.467C > T	p.A156V	-1	-0.26	-0.357	0.0	0.6	A(11)	TIM	No	Yes	[2]
c.496C > G	p.L166	0	-1.18	-0.244	0.0	3.9	B(5)	TIM	No	Yes	[2]
c.548G > C	p.G183A	-2	-0.85	2.330	1.5	5.3	A(18)	TIM	No	Yes	[14]
c.581C > T	p.T194I	-2	-0.57	2.008	76.0	2.6	A(18)	TIM	No	Yes	[43]
c.647A > G	p.Y216C	-3	-1.16	-2.319	11.3	1.9	A(6)	TIM	No	Yes	[14]
c.692A > G	p.D231G	-2	-2.24	-2.726	49.3	66.7	P(5)	TIM	Yes	No	[18]
c.796G > A	p.D266N	-3	-1.30	-0.754	12.8	4.4	O(12)	TIM	No	No	[18]
c.868A > C	p.M290L	0	-0.18	0.486	0.5	2.9	A(14)	TIM	No	Yes	[44]
c.890C > T	p.S297F	-3	-0.73	2.714	0.0	0.0	B(7)	TIM	Yes	No	[18]
c.1118G > A	p.G373D	-3	-0.98	0.251	0.5	0.3	O(12)	beta	No	No	[2]
c.1117G > A	p.G373S	-3	-1.18	-0.256	0.5	0.3	O(12)	beta	No	No	[2]
c.1244T > C	p.L415P	-2	-2.06	-1.232	0.0	0.7	B(9)	beta	No	No	[45]

Mutations not included in the training set of the predictive method are listed with the scores obtained by PSSM (assessment of conservation in homologous sequences), the scores obtained by SDM and MUPRO (assessment of the effect of mutations on thermodynamic stability), % main chain accessibility (PSAmc), % side chain accessibility (PSAsc), occurrence in A alpha-helix, B beta strand, P poly-proline II or O other (with the length of the secondary structure in which the mutation occur in brackets), occurrence in specific structural domains and in the active site. Responsiveness in vitro to 1-deoxy-galactonojirimycin was deduced by literature and the reference paper is reported alongside.

We calculated structural and functional characteristics and the PSSM score for each mutant (table 2) together with the responsiveness to 1-deoxy-galactonojirimycin "in vitro" obtained from literature. As A230T (table 1), three mutations in this set affect the active site and are, invariably, non responsive to 1-deoxy-galactonojirimycin.

This result reinforces the caveat which should always be born in mind when considering PCs for clinical applications: the function of a chaperone is to stabilize the conformational structure of the enzyme, prevent its degradation before it reaches the operative location, but in no case PCs can restore the activity of an enzyme whose active site has been affected by a mutation.

## Conclusions

A surprising high percentage of missense mutations is found in a relatively small protein such as lysosomal alpha galactosidase for reasons that are still unclear. A survey of the list of human proteins associated with diseases provided by Uniprot (http://www.uniprot.org/docs/humpvar release 8_2_2011) provides a flavour of the peculiarity of AGAL_HUMAN and reveals that, excluding tumour suppressors, this protein has the fifth highest ratio between reported missense mutations and chain length; only chain A and B of haemoglobin, Gap junction beta-1 protein (associated with Charcot-Marie-Tooth disease), Transthyretin (associated with amyloidosis type 1) and Phenylalanine-4-hydroxylase (associated with phenylketonuria) have higher rates. The number of missense mutations is likely to increase, since an average of 20-25 novel mutations per year have been identified in the last decade in Fabry disease cases and others are expected from the AGAL mutation screening of the general population.

After genotyping Fabry disease cases, clinicians will face a multitude of different molecular defects and will have to counsel patients about the best therapy to adopt. Enzyme replacement therapy has general applicability, but pharmacological chaperones offer several advantages. This is especially true in the late on-set forms of the disease, when a therapy to prevent the accumulation of glycosphingolipids and hence to delay organ damage, has to be suggested to patients who still have little or no symptoms.

In order to counsel Fabry patients clinicians need to know conditions that are sufficient to make mutations responsive to PC. Regrettably only conditions that are sufficient, but not necessary, to make mutations *non *responsive to PC are clearly identifiable.

By analyzing *in silico *96 mutations, we previously proposed a method that calculates a score based on the construction of PSSM [20]. We have now tested this method on 14 new mutations scoring >-2 and found that 12 (86%) of them were responsive to 1-deoxy-galactonojirimycin. These values differ significantly (Fisher two tailed p value <0.01) from the percentage of mutations (42%) recovering activity (>15% of wild type) in screenings carried out by independent laboratories on randomly collected mutations.

**Figure 4:**
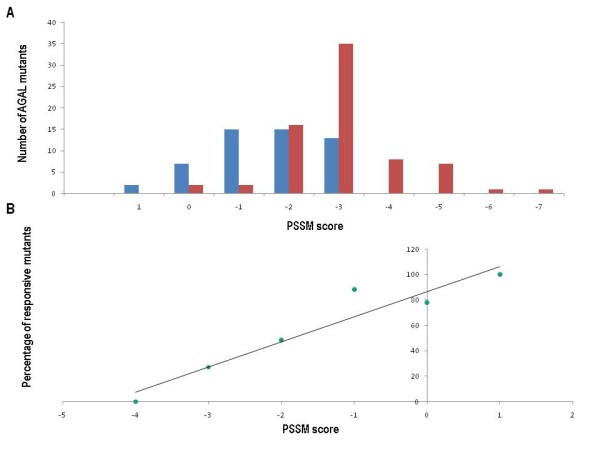
**Distribution of responsive and non responsive alpha-galactosidase mutants and correlation of responsiveness with scores assigned with a Position Specific Substitution Matrix**. Panel A: Distribution of responsive (blue bars) and non responsive (red bars) alpha galactodidase mutants over Position Specific Substitution Matrix scores. Panel B: Percentage of responsive alpha-galactosidase mutants as a function of Position Specific Substitution Matrix scores

In figure 4, panel A, we summarize the results obtained on all AGAL mutants tested "*in vitro*" so far which were responsive to 20 micromolar 1-deoxy-galactonojirimycin(or had an EC50 <20 micromolar). The responsive cases are 86% among the mutations scoring >-2 and only 20% among the mutations scoring <-2 and no responsive cases were observed so far below -3. The correlation between percentage of responsive mutations and PSSM scores in the range +1/-4, is very high and statistically significant, (Pearson correlation coefficient r = 0.96 with two-tailed p-value 0.003) figure 4 panel B.

Our guideline for clinicians is the following: if a new or as-yet untested AGAL mutation has a PSSM score >-2 it is likely to be responsive to 1-deoxy-galactonojirimycin; if it gets a PSSM <-2 it is likely to be non responsive; if it gets PSSM scores -2 it is in a twilight zone where a case by case judgment is necessary. We are aware that a bioassay on cells derived from patients carrying a specific mutation represents a more convincing proof of the efficacy of the treatment with 1-deoxy-galactonojirimycin in any given case. However, we feel that the simplicity and low cost of our method means that it should be considered favorably by clinicians.

## Methods

### Selection and construction of mutants

We collected 13 homologous AGAL sequences from Uniprot/Swissprot [19]; these had E-value <10^-50 ^and sequence identity ranging from 95% to 30%. A Position Specific Substitution Matrix (PSSM) was built from this set of sequences running BLASTPGP (BLASTP2.2.18) in PSI-BLAST mode flagging on the output of a checkpoint file. The PSSM was exploited in a second round of BLASTPGP to calculate net scores for human AGAL mutants [36] obtained by HGMD [7]. Nine mutations were selected among those obtaining score 0 and -1 that had not been previously tested experimentally with 1-deoxy-galactonojirimycin.

The clone SC319065, which contains the full length cDNA for wild type AGAL_HUMAN inserted into the expression vector pCMV6-AC, was purchased from Origene (Rockville, MD, USA). Mutations were introduced with two consecutive PCR reactions. In the first round of amplifications, two reactions were set up, one contained the outmost forward oligo (ECOR1_FORWARD) and the specific reverse mutant oligo (table 3) and the other contained the outmost reverse oligo (XHO1_REVERSE) and the forward specific mutant oligo (table 3). In the second round of amplifications, the purified products of the first PCR reactions were used as templates and the outmost forward and reverse oligos were used as primers. The amplifications were performed for 28 cycles using the following conditions: 95°C for 10 min, 94°C for 30 sec, 60°C for 30 sec, 72°C 30 sec, and 72°C for 5 min with 0.6 μM of each primer. After the second round, amplified fragments were purified, digested with ECORI and XHOI and inserted into pCMV6-AC. Mutant constructs were verified by sequencing.

**Table 3:** Mutated oligonucleotides for site direct mutagenesis

oligo	forward	reverse
ECOR1_RWARD	GCAGAGCTCGTTTAGTGAACCGTCAGAATT	
XHO1_REVERSE		CTGTTCAGGAAACAGCTATGACCGCGGCCG
A230T	GCGAAATTTTACTGACATTGATGATTCCTGG	CAATGTCAGTAAAATTTCGCCAGTGATTGC
E341D	AAGTGTGGGATCGACCTCTCTCAGGCTTAGC	GAGAGGTCGATCCCACACTTCAAAGTTGTCTC
L310F	AGCCAAAGCTTTCCTTCAGGATAAGGACGTA	CCTGAAGGAAAGCTTTGGCTTGAGGGCTGA
V269M	GATATGTTAATGATTGGCAACTTTGGCCTC	TTGCCAATCATTAACATATCTGGGTCATTCC
T410A	ATAAATCCCGCAGGCACTGTTTTGCTTCAG	ACAGTGCCTGCGGGATTTATGTGACTTCTTA
L300F	TCTAATGACTTCCGACACATCAGCCCTCAA	GATGTGTCGGAAGTCATTAGACATGAATAAAG
R301P	TGACCTCCCACACATCAGCCCTCAAGCCA	GGCTGATGTGTGGGAGGTCATTAGACATGAAT
D244H	GTATCTTGCACTGGACATCTTTTAACCAG	GATGTCCAGTGCAAGATACTCTTTATACTTT
Q280K	CTGGAATCAGAAAGTAACTCAGATGGCCCTC	TGAGTTACTTTCTGATTCCAGCTGAGGCCAA

Underlined bases correspond to the mispairings with the normal alpha-galactosidase sequence used to introduce the mutations in the pCMV6-AC-AGAL vector

### Transfection into COS-7 cells

COS-7 were cultured in DMEM containing 10% FBS at 37°C and 5% CO2. The cells were transfected with individual pCMV6-AC plasmids containing wild type or mutant AGAL-encoding ORF using the LipofectAMINE2000 cationic lipid reagent.

Four micrograms of plasmid DNA in 0.5 ml Opti-MEM (Invitrogen) were mixed with 18 microliters LipofectAMINE2000 reagent (Invitrogen) in 0.5 ml Opti-MEM (Invitrogen) and incubated for 20 min at room temperature. During this time, COS-7 cells were harvested by trypsin treatment and resuspended in DMEM containing 10% FBS.

COS-7 in suspension (4 ml) were added to transfection mix solution, distributed into two wells of a six-well plate at 60% confluency and allowed to adhere overnight. The efficiency of transfection was increased by treatment with cloroquine 100 micromolar (SIGMA, Milan, Italy) [14,37]. The efficiency of the transfection was calculated by cotrasfecting an equal amount of pMIR vector (AppliedBiosystem/Ambion, Italy) containing the luciferase gene and assaying the reporter gene activity under standard conditions using ONE-Glo™ Luciferase Assay System (Promega, Italy). A 3-fold increase in efficiency was measured in the presence of cloroquine.

The medium was substituted by fresh DMEM, 10% FBS (3 ml) and in one well 20 micromolar 1-deoxy-galactonojirimycin was added whereas in the other was not. After a 48 hr incubation, the cells were washed in PBS (5 times), scraped and harvested by centrifugation. Dry pellets were resuspended in 200 microliters of water and lysed by freeze-thawing. Four independent transfections were carried out.

Water-soluble extracts were used for enzyme assays or western blot.

### Alpha-galactosidase assay

Cell lysates (2 microliters) were added to 38 microliters of AGAL assay buffer (sodium citrate 27 mM-sodium phosphate dibasic 46 mM, 4-methylumbelliferyl-alpha-D-galactopyranoside 5 mM and N-acetyl-D-galactosamine 100 mM, pH 4.5) and incubated for 1 h at 37°C. All chemicals were obtained from Sigma. The reaction was stopped by adding 360 microliters of 1 M sodium carbonate buffer [15]. Fluorescence was detected using a fluorescence spectrophotometer (Cary Eclypse-Varian) at 355 nm excitation and 460 nm emission.

A 4-methylumbelliferone standard curve ranging from 5 nM to 25 micromolar was run in parallel for conversion of fluorescence data to AGAL activity expressed as nmol/mg protein per min.

### Statistical analysis

Standard deviations and p values in figure 1 (paired two-tailed Student 's t-test) were obtained using Microsoft Excel (Microsoft Office professional 2010).

### Western blot analysis

Western blot analysis for the detection of AGAL was performed by using polyclonal antibody produced in rabbit (Abcam 70520) and HRP-coniugated anti-rabbit IgG antibody produced in goat (Bio-Rad 1706515).

After SDS-PAGE (4-20% acrylamide), proteins were transferred to a PVDF membrane. The membrane was blocked with 5% (w/v) non-fat dried skimmed milk in blot solution at 4°C overnight, then treated with the primary antibody diluted in a blot solution 1:500 for 1 hour at room temperature. After washing with an excess of blot solution, the membrane was treated with the secondary antibody diluted in the blot solution 1:2000 for 1 hour at room temperature. After washing, the detection was performed by using the Immun-Star WesternC chemiluminescence detection kit (Bio-Rad). GAPDH was revealed using with mouse monoclonal anti lapine GAPDH (AbD seroTec (cat:4699-9555) 1:20000. Fabrazyme^® ^(agalsidase-beta; Genzyme Europe B. V., NL). All other chemicals were from Bio-Rad.

### In silico characterization

Models of all AGAL mutants are constructed using PDB file 3GXT as template [33]. Side chains were substituted with the program ANDANTE, which predicts side-chain conformations by use of environment-specific substitution probabilities and a high-quality rotamer library [38].

SDM [22,23] uses a set of conformationally constrained environment-specific substitution tables and calculates the difference in the stability scores for the folded and unfolded state of each mutant and the wild-type protein. It uses as inputs the mutants generated with ANDANTE and the structure of the wild type enzyme.

Residue percent accessibility in AGAL dimer structure (3GXT) was calculated with PSA v2.0 [39] which uses the rolling probe algorithm [40]. We assigned each residue in the wild type AGAL structure 3GTX to alpha helices, beta sheets, poly-proline helix or other with the program SEGNO [35].

MUPRO1.1 was run locally, using as inputs only AGAL sequence, the position affected by the mutation, the original and the mutant amino-acid.

The programs SDM [22,23], ANDANTE and PSA were kindly provided by T. Blundell and collaborators. MUPRO1.1 [21] was kindly provided by Dr J. Cheng.

## Abbreviations

DGJ: Deoxy-galactonojirimycin; FD: Fabry disease; MUPRO: Prediction of Protein Stability Changes for Single-Site Mutations from Sequences; PSA: Protein Solvent Accessibility; PC: pharmacological chaperone; PSSM: position specific substitution matrix, SDM: Site Directed Mutator; TIM: triosephosphate isomerase-like domain.

## Competing interests

The authors declare that they have no competing interests.

## Authors' contributions

GA and MVC designed the study and wrote the paper. VC carried out transfections, ADC and AC carried out mutagenesis, GA carried out enzyme assays. MC carried out structure based and sequence based analysis, PO and GA carried out western blots. All authors read and approved the final manuscript
